# Cardiovascular syphilis: a rare case report and literature review of severe aortic regurgitation and bilateral coronary ostial stenosis

**DOI:** 10.3389/fcvm.2025.1444974

**Published:** 2025-08-25

**Authors:** Ru Wang, Li Zhang, Binfeng Xia, Min Kong, Huihui Huang, Xia Yin

**Affiliations:** Department of Cardiovascular Medicine, The First Hospital of Jilin University, Changchun, China

**Keywords:** cardiovascular syphilis, bilateral coronary ostial stenosis, severe aortic regurgitation, heart failure, differential diagnosis

## Abstract

Due to the low incidence of cardiovascular involvement in syphilis, there are currently no established consensus or guidelines for managing such cases. The patient, with no coronary artery disease risk factors, presented with chest pain and heart failure (HF). Emergency coronary angiography revealed severe stenosis of the bilateral coronary ostia, with smooth intimal lining in the remaining coronary arteries. Echocardiography showed severe aortic regurgitation. Positive syphilis serology strongly suggested cardiovascular syphilis (CVS). The patient was transferred to cardiothoracic surgery for aortic valve replacement (AVR) and coronary artery bypass grafting (CABG), but died from acute HF despite resuscitation efforts.

## 1 Introduction

CVS is a rare but fatal late complication (1), with incidence resurging globally due to factors (2) such as changes in sexual behavior, increased HIV prevalence, and inadequate public health resources. Clinical manifestations typically emerge 10–30 years after infection (3), making diagnosis challenging, especially in elderly patients with cardiovascular risk factors. CVS lesions often mimic atherosclerotic changes (4), leading to misdiagnosis or delayed diagnosis. This case report describes a rare instance of CVS involving both the aortic valve and bilateral coronary ostial stenosis, presenting with chest pain similar to coronary artery disease, complicating differential diagnosis. Mistakes in treatment (incorrect selection of anti-syphilitic drugs and limited circulatory support strategies) highlight key clinical pitfalls. By comparing this case with similar reported cases, we aim to aid clinicians in early and accurate identification of critical CVS, ensuring timely intervention.

## 2 Case presentation

**Figure 1:**
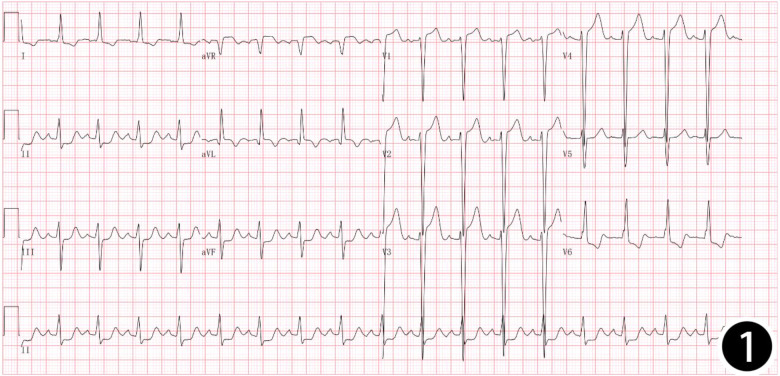
12-lead: sinus tachycardia, left ventricular hypertrophy, poor R-wave progression in the precordial leads, and ST-T changes.

**Figure 2:**
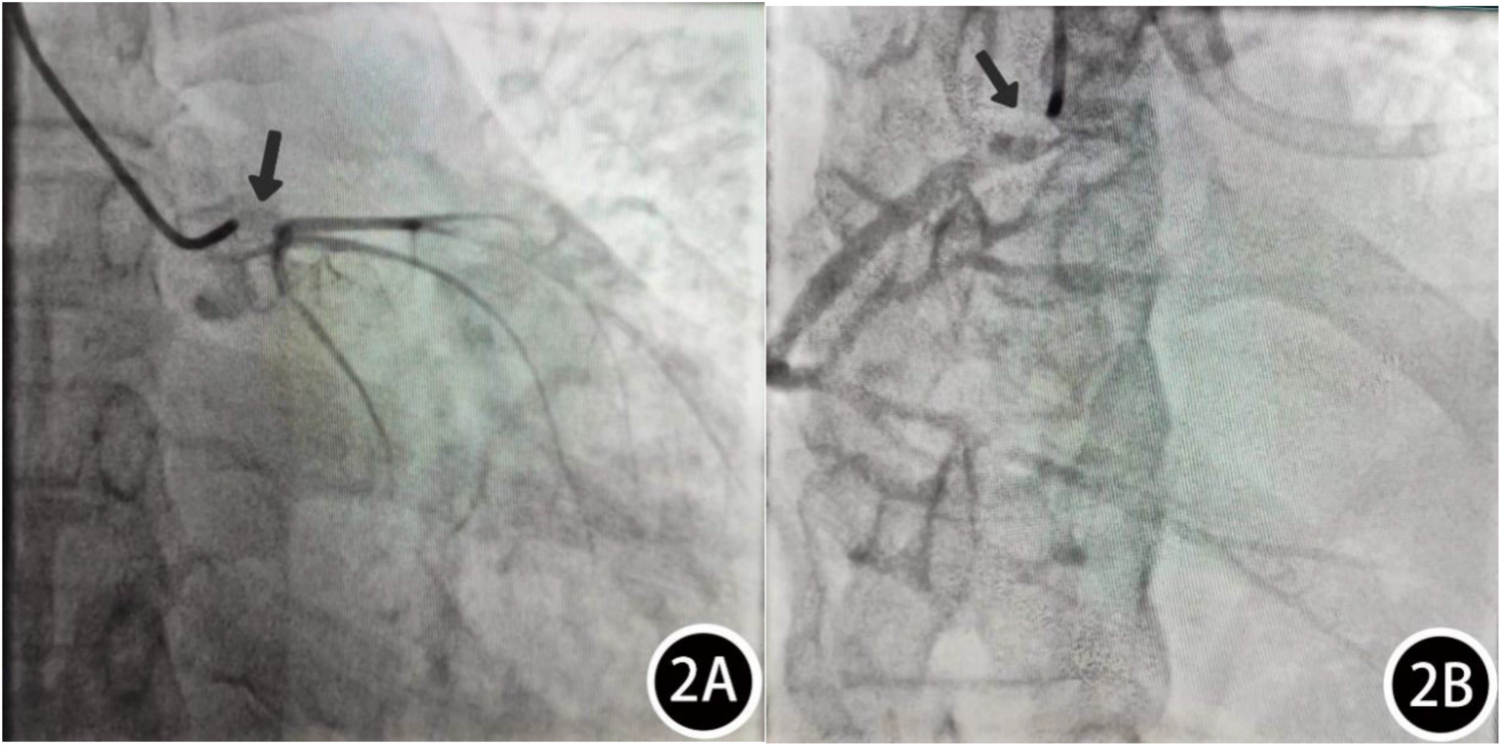
Coronary angiography results: **(A)** LM: 80% ostial stenosis. **(B)** RCA: 90% ostial stenosis.

**Figure 3:**
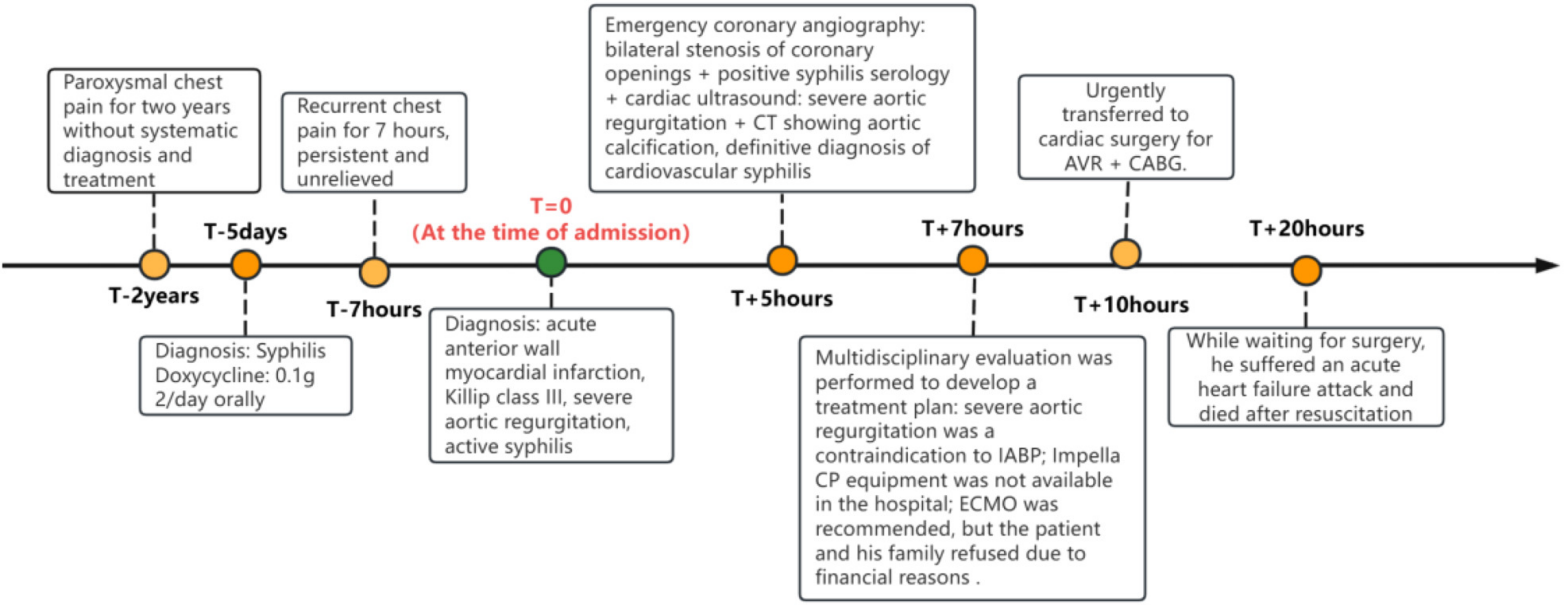
A timeline of all the patient's events.

Patient, female, 45 years old, was admitted with “paroxysmal chest pain for more than 2 years, and recurrent chest pain for 7 h.” Two years ago, the patient began experiencing retrosternal pain during physical activity, with an unclear nature and variable duration, and did not seek systemic treatment. Seven hours before admission, she again experienced retrosternal burning pain without radiation, persistent and unrelieved, accompanied by chest tightness and shortness of breath. She presented to the emergency department (ED), where an electrocardiogram (ECG) showed “acute anterior myocardial infarction.” She was subsequently admitted to the coronary care unit (CCU). Five days prior, the patient was diagnosed with “syphilis.” Due to a penicillin allergy, she was currently taking doxycycline 0.1 g twice daily. Her medical history was otherwise unremarkable. On admission, her vital signs were as follows (Table 1): temperature 36.5°C, respiration rate 18 breaths/min, blood pressure 100/70 mmHg, alert and oriented, no jugular venous distention, bilateral lung bases revealed coarse rales, apex beat located 0.5 cm lateral to the left midclavicular line in the 5th intercostal space, heart rate 75 bpm with regular rhythm, a diastolic murmur with a “gallop” sound was audible at the second aortic auscultation area, no edema in the lower extremities. Initial lab results after admission were as follows: Troponin I (cTnI) 0.173 ng/ml (reference value: 0–0.034 ng/ml), creatine kinase myocardial band (CK-MB) 1.75 ng/ml (reference value: 0–3.38 ng/ml), Myoglobin (Mb) 65.8 ng/ml (reference value: 0–61.5 ng/ml), brain natriuretic peptide(BNP) 1,580.0 pg/ml (reference value: 0–100 pg/ml), Treponema pallidum antibodies (TP-Ab) 23.49 S/CO, Rapid Plasma Reagin Test (RPRT) 1:128 positive. Rheumatological and immunological tests were unremarkable, and other laboratory results were within normal limits. The ECG showed (Figure 1): sinus tachycardia, left ventricular hypertrophy, poor R-wave progression in the precordial leads, and ST-T changes. Bedside echocardiography showed (Table 2): aortic root diameter 38 mm, left atrial diameter 37 mm, right ventricular diameter 19 mm, interventricular septum thickness 7 mm, left ventricular end-diastolic diameter 61 mm, left ventricular posterior wall thickness 7 mm, inferior vena cava diameter 20 mm with 50% collapse. Cardiac function: Ejection fraction (EF) 28%, End-diastolic volume (EDV) 215 ml, End-systolic volume (ESV) 154 ml, Stroke volume (SV) 61 ml, Cardiac output (CO) 6.22 L/min, Heart rate (HR) 103 bpm. Doppler echocardiography showed: mitral valve flow, E wave 89 cm/s, A wave 67 cm/s; pulmonary valve peak flow velocity 61 cm/s. Tissue Doppler Imaging (TDI): E/e′ 8–14. Left atrial and ventricular diameters were increased. The interventricular septum and left ventricular posterior wall thickness were normal. Diffuse hypokinesia of the left ventricle was noted. The aortic valve showed increased echoes, thickened and unclear leaflets. Doppler echocardiography of the aortic valve showed a peak velocity of 252 cm/s, a Pressure gradient (PG) of 23 mmHg. Color Doppler Flow Imaging (CDFI) showed an aortic regurgitation area of 13.0 cm^2^, with a vena contracta of 9 mm. CDFI also showed mitral regurgitation area of 8.2 cm^2^ and tricuspid regurgitation area of 7.2 cm^2^, with a maximum regurgitant velocity of 383 cm/s and PG 58 mmHg, estimating a pulmonary artery systolic pressure of 64 mmHg. Pulmonary artery valve regurgitation area was 3.6 cm^2^, with a maximum velocity of 261 cm/s and PG 27 mmHg. Chest CT showed: bronchitis, scattered inflammation in both lungs, thickening of the interlobular septa, possible interstitial pulmonary edema, bilateral pleural effusions, and atherosclerosis of the thoracic aorta. Based on the patient's symptoms, signs, and auxiliary tests, acute myocardial infarction was diagnosed. After oral administration of 300 mg aspirin and 300 mg clopidogrel, an emergency coronary angiography was performed. The results (Figure 2) showed: Left Main Coronary Artery (LM): 80% ostial stenosis; Left Anterior Descending Artery (LAD): no significant stenosis observed; TIMI grade 3 flow was maintained; Left Circumflex Artery (LCX): no significant stenosis observed; TIMI grade 3 flow was maintained; Right Coronary Artery (RCA): 90% ostial stenosis, TIMI grade 3 flow was maintained. There was no collateral circulation. The dominant coronary artery was the right dominant type. Given the coronary angiography findings, echocardiography, and positive syphilis serology, the primary surgeon determined that the acute myocardial infarction was not due to atherosclerosis but was instead related to syphilitic heart disease. The patient and family were informed of the current diagnosis: CVS, acute extensive anterior myocardial infarction, severe aortic regurgitation, Killip class III. The patient's vital signs remained unstable, with imminent risk of sudden death due to acute HF. The patient presented with severe aortic regurgitation (AR), with a regurgitant area of 13 cm^2^. Intra-aortic balloon pump (IABP) placement would increase diastolic pressure by 20%–30%, thereby increasing regurgitant volume by ≥30%, exacerbating left ventricular volume overload, and accelerating the development of pulmonary edema. According to the 2021 ESC guidelines, IABP is contraindicated in patients with AR (Class III recommendation). Our institution does not have access to the Impella CP device, and obtaining it from external sources would require more than 12 h. Percutaneous coronary intervention (PCI) for coronary ostial stenosis caused by CVS is associated with a 100% rate of re-occlusion after stent placement. Veno-arterial extracorporeal membrane oxygenation (VA-ECMO) was recommended to provide cardiopulmonary support, reduce cardiac workload, and ensure adequate coronary perfusion, serving as a bridge to definitive surgical intervention. However, due to financial constraints, the patient and family declined ECMO treatment and agreed to transfer to the cardiothoracic surgery department for AVR and CABG. The patient remained in poor general condition, maintained in a semi-recumbent position. At approximately 2:00 AM the following day, the patient developed agitation, profuse sweating, cold extremities, and dyspnea. Immediate cardiopulmonary resuscitation (CPR), inotropic support, endotracheal intubation, and electrical defibrillation were administered. Despite aggressive resuscitative efforts, the patient ultimately succumbed to circulatory collapse and died (Figure 3).

**Table 1:** Physical examination and laboratory data.

Parameter	Values	Reference value	Unit
Temperature	36.5	36.0–37.0	°C
Blood pressure	100/70	90/60–140/90	mmHg
Heart rate	75	60–100	times/min
Pulse	75	60–100	times/min
Respiratory frequency	18	12–20	times/min
TnI	0.173	0–0.034	ng/ml
CK–MB	1.75	0–3.38	ng/ml
Mb	65.8	0–61.5	ng/ml
BNP	1,580	0–100	pg/ml
TP–Ab	23.49	—	S/CO
RPRT	1:128 positive	—	—

cTnI, troponin I; CK-MB, creatine kinase myocardial band; Mb, myoglobin; BNP, brain natriuretic peptide; TP-Ab, Treponema pallidum antibody; RPRT, rapid plasma regain test.

**Table 2:** Bedside cardiac ultrasound data.

Category	Item	Measurement/Result
Cardiac Structure Measurements	Ascending aortic diameter	38 mm
	Left atrial anteroposterior diameter	37 mm
	Right ventricular anteroposterior diameter	19 mm
	Interventricular septal thickness	7 mm
	Left ventricular end-diastolic diameter	61 mm
	Left ventricular posterior wall thickness	7 mm
	Inferior vena cava diameter	20 mm
	Inferior vena cava collapse rate	50%
Cardiac Function Parameters	EF	28%
	EDV	215 ml
	ESV	154 ml
	SV	61 ml
	CO	6.22 L/min
	HR	103 bpm
Doppler Flow Parameters	Mitral valve E wave	89 cm/s
	Mitral valve A wave	67 cm/s
	Maximum pulmonary valve flow velocity	61 cm/s
TDI	E/e′	8–14
CDFI Regurgitation	Maximum velocity above aortic valve	252 cm/s, PG: 23 mmHg
	Aortic valve regurgitation area	13.0 cm^2^; Vena contracta: 9 mm
	Mitral valve regurgitation area	8.2 cm^2^
	Tricuspid valve regurgitation area	7.2 cm^2^
	Maximum tricuspid regurgitation velocity/PG	383 cm/s, PG: 58 mmHg
	Estimated pulmonary artery systolic pressure	64 mmHg
	Pulmonary valve regurgitation area	3.6 cm^2^
	Maximum pulmonary valve regurgitation velocity/PG	261 cm/s, PG: 27 mmHg

EF, ejection fraction; EDV, end-diastolic volume; ESV, end-systolic volume; SV, stroke volume; CO, cardiac output; HR, heart rate; PG, pressure gradient; TDI, tissue doppler imaging; CDFI, color doppler flow imaging.

## 3 Discussion

### 3.1 Epidemiology and mechanism

Syphilis is a chronic systemic infection caused by Treponema pallidum, classically divided into primary, secondary (early), and tertiary (late) stages. Because primary syphilis can be asymptomatic and may resolve spontaneously, many patients do not receive adequate treatment. Approximately 30% of patients with untreated early syphilis progress to tertiary syphilis 10–30 years after initial infection. This late stage can involve multiple organ systems and present as gummatous syphilis, neurosyphilis, or CVS (3). Among these, around 25% of tertiary syphilis cases are estimated to develop cardiovascular involvement (5). CVS encompasses five main pathological entities: syphilitic aortitis, syphilitic aortic regurgitation, syphilitic aortic aneurysm, syphilitic coronary artery stenosis, and syphilitic gummas of the myocardium (6). Syphilitic aortitis represents the pathological basis of CVS. After penetrating through mucocutaneous lesions, T. pallidum enters the bloodstream and invades the vasa vasorum in the adventitia of the aorta. This leads to obliterative endarteritis, causing necrosis of elastic fibers and smooth muscle cells in the media, resulting in thinning and dilation of the aortic wall, and the characteristic “eggshell” calcification. When the inflammation involves the aortic root, it can cause annular dilatation and resultant aortic valve insufficiency. If it affects the mid-ascending aorta, contraction of fibrotic tissue may lead to concentric stenosis of the coronary ostia. Syphilitic aortitis has been reported to result in aortic aneurysms in 71% of cases, aortic regurgitation in 47%, and coronary ostial stenosis in approximately 16.5% of cases (7, 8). Among these, bilateral coronary ostial stenosis is considered a particularly rare manifestation of syphilitic aortitis.

### 3.2 Diagnosis and differential diagnosis

The gold standard for diagnosing CVS is the histopathological identification of Treponema pallidum and the presence of obliterative endarteritis of the vasa vasorum. However, because aortic biopsy is rarely performed in clinical practice, this method is largely restricted to selected challenging cases. According to the 2023 WHO guidelines, clinical diagnosis of CVS should be based on a combination of the following: positive T. pallidum-specific serological tests, imaging evidence of aortitis, and exclusion of alternative diagnoses. This case represents a classic manifestation of tertiary syphilis involving the cardiovascular system, with a rare and life-threatening combination of severe aortic regurgitation and bilateral coronary ostial stenosis. The diagnosis was supported by the following key findings: 1. Strongly positive syphilis serology (TPPA: 23.49 S/CO; RPR: 1:128); 2. Evidence of aortitis on imaging (dilated ascending aorta measuring 38 mm, aortic wall calcification, thickened valve leaflets, and severe aortic regurgitation); 3. Exclusion of atherosclerosis, based on absence of traditional coronary artery disease risk factors, and angiographic findings of isolated ostial stenosis (80% stenosis at the left main ostium, 90% at the right coronary ostium) with otherwise smooth and normal distal coronary arteries; 4. Exclusion of rheumatic or autoimmune diseases, supported by normal immunological and inflammatory markers.

Differential Diagnosis of CVS: The pathophysiological basis of CVS is distinct from that of coronary atherosclerosis. Atherosclerotic coronary artery disease is characterized by the development of atheromatous plaques composed of lipids, inflammatory cells, and connective tissue, which progressively narrow the coronary artery lumen. In contrast, syphilitic coronary stenosis results from obliterative endarteritis with chronic inflammatory infiltration, ischemic necrosis, and medial fibrosis. The contraction of fibrotic scar tissue leads to isolated stenosis at the coronary ostia (9), rather than diffuse atherosclerotic plaque formation along the entire coronary artery (10). While atherosclerosis is a common cause of coronary artery stenosis, it typically occurs in the presence of traditional cardiovascular risk factors such as hypertension and hyperlipidemia. Rheumatic heart disease may also cause aortic valve pathology, but it is usually associated with a history of rheumatic fever. Infective endocarditis may result in valvular destruction, yet it is often accompanied by systemic signs such as fever and bacteremia. Additionally, large-vessel vasculitis may involve the aorta and its branches, but it is generally associated with clinical and laboratory features of autoimmune disease.

Coronary artery stenosis caused by CVS typically develops slowly and is often accompanied by the formation of collateral circulation (11), which makes large myocardial infarctions rare. Clinically, patients may remain asymptomatic or present with symptoms mimicking acute coronary syndrome due to progressive medial fibrosis of the ascending aorta extending to the coronary ostia, resulting in ostial stenosis (12).In rare cases, initiation of antisyphilitic treatment can lead to a JHR—a hypersensitivity response that occurs 24–48 h after treatment when Treponema pallidum is rapidly killed and releases a large quantity of antigenic proteins. This reaction can induce swelling at the coronary ostia, aggravate stenosis, and even cause complete coronary ostial occlusion, potentially resulting in sudden cardiac death. Echocardiography plays an important role in the early diagnosis of CVS. Key early sonographic features include dilation of the aortic root and ascending aorta, thickening of the aortic wall, and increased valvular thickness (13). Additionally, experienced interventional cardiologists may recognize angiographic patterns suggestive of non-atherosclerotic stenosis. In clinical practice, when patients present with chest pain but lack conventional coronary artery disease risk factors, and coronary angiography reveals isolated coronary ostial stenosis with smooth distal vessel walls, CVS should be strongly suspected. Conversely, the presence of additional stenotic lesions beyond the coronary ostia increases the likelihood of atherosclerotic coronary artery disease (14).

### 3.3 Characteristics of this case and reflections on treatment

This case differs from previously reported instances in its rare and critical combination of bilateral coronary ostial stenosis and severe aortic regurgitation (AR). Diagnostic and therapeutic pitfalls in this case provide important clinical lessons in managing life-threatening CVS. The first major error was the continued use of oral doxycycline after CVS diagnosis. As clearly emphasized by the WHO guidelines (2023), doxycycline predominantly distributes in soft tissues. It has poor penetration into the fibrotic vascular walls of CVS due to the presence of obliterative endarteritis, which acts as a pharmacologic barrier. Consequently, doxycycline fails to achieve adequate local antibiotic concentrations, cannot induce sufficient treponemal lysis, and is ineffective against CVS. In penicillin-allergic patients such as this one, doxycycline should have been promptly replaced with intravenous ceftriaxone (2 g IV daily), initiated in combination with prophylactic corticosteroids (e.g., prednisone 20 mg daily for 3 days) to prevent a JHR. Notably, the patient had already taken doxycycline for five days before experiencing persistent chest pain, which makes JHR (typically occurring within 24–48 h) an unlikely cause of symptom exacerbation. The ongoing chest pain is more likely to reflect the natural progression of coronary ostial stenosis. The timing of JHR depends on the rate of treponemal lysis and is independent of the antibiotic class. The second critical misstep was the absence of effective circulatory support in the context of acute HF (Killip Class III) induced by bilateral coronary ostial stenosis and severe AR. In this setting, intra-aortic balloon pump (IABP) is contraindicated due to severe aortic regurgitation (ESC 2021, Class III recommendation). Our center lacked access to Impella devices, and due to financial constraints, the patient and family declined VA-ECMO. As a result, advanced circulatory support strategies such as ECMO or ECPELLA (ECMO + Impella CP) could not be implemented to stabilize hemodynamics, unload the left ventricle, and gain time for surgical intervention. This outcome highlights the critical importance of resource availability and timely decision-making in the management of fulminant CVS. The patient ultimately succumbed to hemodynamic collapse while awaiting surgery. The presence of severe aortic regurgitation (regurgitant area: 13 cm^2^) likely contributed to myocardial ischemia through three synergistic mechanisms: First, AR led to a sharp decline in diastolic coronary perfusion pressure, reduced antegrade flow, and decreased effective stroke volume, all contributing to impaired myocardial oxygen supply. Second, left ventricular volume overload and dilation increased wall stress and heart rate (up to 103 bpm), raising myocardial oxygen demand. However, coronary ostial stenosis restricts oxygen delivery, creating a supply-demand mismatch. Third, elevated left ventricular end-diastolic pressure (LVEDP) directly compressed subendocardial coronary vessels, particularly affecting the anterior wall. These mechanisms interacted to form a vicious cycle: AR reduced effective stroke volume, further limiting coronary perfusion and exacerbating ischemia and left ventricular dysfunction, eventually culminating in transmural myocardial infarction.

### 3.4 Literature review and treatment plan

The pathophysiological basis of coronary artery stenosis in CVS is distinct from that of atherosclerotic coronary artery disease (CAD), and therefore, the treatment strategy for syphilitic coronary stenosis differs from that for atherosclerotic disease. In cases of coronary ostial stenosis caused by syphilis, CABG or endarterectomy was historically advocated. However, with the advancement of PCI techniques, improvements in interventional equipment, and progress in pharmacotherapy, PCI has emerged as an effective treatment option for selected patients with CVS-related coronary stenosis (15). Xiangdong Li et al. (16) emphasized that unprotected PCI for left main coronary artery lesions is contraindicated due to the high risk of catastrophic complications. For coronary ostial stenosis, PCI under ECMO support has been shown to ensure procedural success while reducing surgical risk (17). Fang Haiyang et al. (18) suggested that in cases of bilateral coronary ostial stenosis complicated by cardiogenic shock, intra-aortic balloon pump (IABP) should be first inserted to stabilize hemodynamics, followed by emergency PCI of the left main lesion. Once cardiac function improves, the right coronary lesion can be addressed subsequently, resulting in favorable short-term outcomes. Zhang Xiaorong et al. (19) proposed that in patients with HF, antisyphilitic therapy should be delayed until HF is adequately controlled. Treatment should start with low-dose penicillin and be escalated gradually. In cases with progressive dyspnea, myocardial infarction, severe conduction disturbances, or large aortic aneurysms, immediate antisyphilitic therapy is not recommended, as improper treatment may accelerate clinical deterioration or even lead to death. Tamer Ghazy et al. (20) reported a case of fatal aortic rupture following penicillin treatment for a syphilitic aortic aneurysm. One of the main challenges of PCI for syphilitic coronary ostial stenosis is restenosis, as well as the inability to address concurrent vascular complications such as aortic regurgitation or aneurysm. For isolated coronary ostial stenosis, current evidence suggests that CABG remains the most effective treatment modality (9, 21, 22). A recent 4-year retrospective study demonstrated that the incidence of restenosis at 6 months was significantly lower in patients treated with CABG compared to those who underwent PCI (21). The last 2 years of research have shown that (23–25) “Open” stent placement adds an option to the armamentarium of open-heart surgery for isolated ostial stenosis of the left (or right) coronary trunks. Long-term and regular follow-up is valuable for the treatment of such diseases.

Systematic Treatment Strategy for CVS: Emergency Management Phase: a. Circulatory Support: For patients with aortic regurgitation, mechanical circulatory support should prioritize Impella 5.0 (*via* surgical cutdown) or Impella CP combined with ECMO (ECPELLA) to provide adequate left ventricular unloading. b. Management of Acute Myocardial Infarction (AMI): Initiate dual antiplatelet therapy, avoid heparin anticoagulation, and use nitrates with caution due to the risk of exacerbating hypotension in the setting of severe aortic regurgitation. Intensified Antisyphilitic Therapy: The first-line treatment is aqueous penicillin G; for penicillin-allergic patients, ceftriaxone is recommended. Preventive measures against the JHR should be implemented, and therapeutic efficacy should be monitored through clinical and serological follow-up. Intervention for Cardiovascular Lesions: (a) Surgical Indications and Timing: Emergency Surgery: Indicated for aortic aneurysms >5 cm or impending rupture, to be performed within 24 h. Urgent Surgery: Indicated for severe aortic regurgitation with EF <35% or coronary ostial stenosis >70%, recommended within 3–7 days. Elective Surgery: In hemodynamically stable patients, surgery may be scheduled after completion of 14 days of antisyphilitic therapy. (b) Surgical Strategy: A comprehensive surgical approach may include AVR, CABG, and the Bentall procedure, depending on the extent of cardiovascular involvement. Postoperative Management: Completion of a full 21-day course of antisyphilitic therapy. Gradual weaning from ECMO and other mechanical support. Regular serological follow-up to monitor for treatment response and detect recurrence.

## 4 Conclusions

1. The resurgence of syphilis worldwide necessitates early recognition and standardized treatment. The global incidence of syphilis is rising, underscoring the importance of early diagnosis and adequate antibiotic therapy to prevent late-stage cardiovascular complications. Comprehensive sexual history-taking and routine serologic screening should be emphasized to raise awareness among healthcare providers and patients alike.
2. CVS should be considered in ischemic chest pain patients without traditional coronary risk factors. In patients presenting with chest pain and echocardiographic findings such as aortic root dilation or valvular thickening—especially in the absence of conventional atherosclerotic risk factors—CVS should be actively investigated. Seronegativity does not definitively rule out the diagnosis.
3. Caution is required when initiating antisyphilitic therapy due to the risk of JHR. In unstable patients with suspected coronary ostial stenosis, antisyphilitic therapy should not be initiated without first assessing coronary anatomy. If indicated, treatment should begin under the cover of low-dose corticosteroids to reduce the risk of JHR, which may exacerbate coronary inflammation and precipitate life-threatening occlusion. Close clinical monitoring is essential during the initial treatment phase.
4. This case highlights two critical pitfalls and offers insight into optimal management of CVS. In patients presenting with acute myocardial infarction, severe aortic regurgitation, and isolated bilateral coronary ostial stenosis, CVS must be considered. Missteps such as the inappropriate use of doxycycline and contraindicated circulatory support (e.g., IABP in aortic regurgitation) should be avoided. Prompt initiation of effective antisyphilitic therapy (penicillin or ceftriaxone with corticosteroid prophylaxis) and advanced circulatory support (e.g., ECMO or Impella) are essential to stabilize the patient and create a surgical window.

## 5 Shortcomings

Based on previously reported similar cases, international guidelines, and the latest evidence-based recommendations, the following deficiencies were identified in the management of this case:

1. Inadequate Antisyphilitic Therapy: Following the diagnosis of CVS, the patient should have been promptly transitioned from oral doxycycline to intravenous ceftriaxone to achieve higher antimicrobial concentrations and improve therapeutic efficacy against Treponema pallidum in fibrotic vascular tissues.
2. Delayed Hemodynamic Support: Simultaneous initiation of ECMO combined with left ventricular unloading is essential in CVS patients with acute decompensated HF. However, due to the unavailability of the Impella device at our center and the patient's/family's refusal of ECMO therapy, progressive hemodynamic collapse and ongoing myocardial ischemia ensued. IABP is contraindicated in the setting of severe aortic regurgitation. Early adjustment of the antisyphilitic regimen in combination with timely initiation of ECMO and left ventricular unloading could have stabilized hemodynamics, allowed time for surgical intervention, and potentially improved prognosis and survival in such critically ill patients.
3. Lack of Histopathological Confirmation: The patient and family declined surgical biopsy for histopathological examination of syphilitic aortitis or coronary ostial stenosis, which precluded definitive pathological confirmation of CVS-related vascular lesions.

## Data Availability

The original contributions presented in the study are included in the article/Supplementary Material, further inquiries can be directed to the corresponding author.
