# Supplementary material for: Cardiovascular syphilis: a rare case report and literature review of severe aortic regurgitation and bilateral coronary ostial stenosis
Source: Front Cardiovasc Med. 2025 Aug 25;12:1444974. doi: 10.3389/fcvm.2025.1444974 (PMC12414935; doi:10.3389/fcvm.2025.1444974)
Supplement: Supplementary file 1 [file Datasheet1.pdf]

CARE case report guidelines

CARE Checklist of information to include when writing a case report

(cc) BY-NC-ND

Topic	Item	Checklist item description	Reported on Line
Title	1	The diagnosis or intervention of primary focus followed by the words "case report"	Title
Key Words	2	2 to 5 key words that identify diagnoses or interventions in this case report, including "case report"	Key words
Abstract (no references)	3a	Introduction: What is unique about this case and what does it add to the scientific literature?	Introduction
	3b	Main symptoms and/or important clinical findings	Case presentation
	3c	The main diagnoses, therapeutic interventions, and outcomes	Case presentation
	3d	Conclusion—What is the main "take-away" lesson(s) from this case?	Conclusions
Introduction	4	One or two paragraphs summarizing why this case is unique (**may include references**)	Introduction
Patient Information	5a	De-identified patient specific information.	Case presentation
	5b	Primary concerns and symptoms of the patient.	Case presentation
	5c	Medical, family, and psycho-social history including relevant genetic information	Case presentation
	5d	Relevant past interventions withoutcomes	Case presentation
Clinical Findings	6	Describe significant physical examination (PE) and important clinical findings.	Case presentation
Timeline	7	Historical and current information from this episode of care organized as a timeline	Figure 3
Diagnostic Assessment	8a	Diagnostic testing (such as PE, laboratory testing, imaging, surveys).	Case presentation
	8b	Diagnostic challenges (such as access to testing, financial, or cultural)	Discussion:2.Diagnosis and differential diagnosis
	8c	Diagnosis (including other diagnoses considered)	Discussion:2.Diagnosis and differential diagnosis
	8d	Prognosis (such as staging in oncology) where applicable	NO
Therapeutic Intervention	9a	Types of therapeutic intervention (such as pharmacologic, surgical, preventive).	Discussion:4.Literature review and treatment plan
	9b	Administration of therapeutic intervention (such as dosage, strength, duration)	Discussion:4.Literature review and treatment plan
	9c	Changes in therapeutic intervention (with rationale)	Discussion:4.Literature review and treatment plan
Follow-up and Outcomes	10a	Clinician and patient-assessed outcomes (if available)	NO
	10b	Important follow-up diagnostic and other test results	NO
	10c	Intervention adherence and tolerability (How was this assessed?)	NO
	10d	Adverse and unanticipated events	Discussion:Characteristics of this case and reflections on treatment
Discussion	11a	A scientific discussion of the strengths AND limitations associated with this case report	Discussion:Characteristics of this case and reflections on treatment; Shortcomings
	11b	Discussion of the relevant medical literature **with references**.	Discussion:Literature review and treatment plan; References
	11c	The scientific rationale for any conclusions (including assessment of possible causes)	Discussion and References
	11d	The primary "take-away" lessons of this case report (without references) in a one paragraph conclusion	Conclusions
Patient Perspective	12	The patient should share their perspective in one to two paragraphs on the treatment(s) they received	Case presentation
Informed Consent	13	Did the patient give informed consent? Please provide if requested	Yes ☐ No ☑
